# The Mixed Role of Sleep and Time of Day in Working Memory Performance of Older Adults with Mild Cognitive Impairment

**DOI:** 10.3390/healthcare12161622

**Published:** 2024-08-14

**Authors:** Michael Georgoudas, Despina Moraitou, Eleni Poptsi, Emmanouil Tsardoulias, Despina Kesanli, Vasileios Papaliagkas, Magda Tsolaki

**Affiliations:** 1 IPPS “Neuroscience and Neurodegeneration”, Faculty of Medicine, Aristotle University of Thessaloniki (AUTh), 54124 Thessaloniki, Greece; 2 Laboratory of Psychology, Department of Cognition, Brain and Behavior, School of Psychology, Aristotle University of Thessaloniki (AUTh), 54124 Thessaloniki, Greece; demorait@psy.auth.gr (D.M.); poptsielena@gmail.com (E.P.); 3 Laboratory of Neurodegenerative Diseases, Center for Interdisciplinary Research and Innovation, Aristotle University of Thessaloniki (CIRI-AUTh), 54124 Thessaloniki, Greece; tsolakim1@gmail.com; 4 Day Center “Greek Association of Alzheimer’s Disease and Related Disorders (GAADRD)”, 54643 Thessaloniki, Greece; 5 School of Electrical and Computer Engineering, Faculty of Engineering, Aristotle University of Thessaloniki (AUTh), 54124 Thessaloniki, Greece; etsardou@ece.auth.gr; 6 School of Psychology, Faculty of Philosophy, Aristotle University of Thessaloniki (AUTh), 54124 Thessaloniki, Greece; despina.kesanli@yahoo.gr; 7 Department of Biomedical Sciences, International Hellenic University, 57001 Thessaloniki, Greece; vpapaliagkas@gmail.com

**Keywords:** aging, Alzheimer’s disease (AD), working memory storage, working memory processing, working memory updating

## Abstract

The importance of night sleep for maintaining good physical and cognitive health is well documented as well as its negative changes during aging. Since Mild Cognitive Impairment (MCI) patients bear additional disturbances in their sleep, this study aimed at examining whether there are potential mixed effects of sleep and afternoon time of day (ToD) on the storage, processing, and updating components of working memory (WM) capacity in older adults with MCI. In particular, the study compared patients’ performance in the three working memory components, in two-time conditions: “early in the morning and after night sleep”, and “in the afternoon and after many hours since night sleep”. The Working Memory Capacity & Updating Task from the R4Alz battery was administered twice to 50 older adults diagnosed with MCI. The repeated measures analysis showed statistically significant higher performance in the morning condition for the working memory updating component (*p* < 0.001). Based on the findings, it seems that the afternoon ToD condition negatively affects tasks with high cognitive demands such as the WM updating task in MCI patients. These findings could determine the optimal timing for cognitive rehabilitation programs for MCI patients and the necessary sleep duration when they are engaged in cognitively demanding daily activities.

## 1. Introduction

The aging population has been rapidly growing all over the world. People aged 65 and over, comprising 10% of the worldwide population in 2020, account for around 720 million people [1]. This population is expected to rise to 16% by 2050 [2]. In this age group, studies have shown that the geriatric syndromes of frailty, sarcopenia, weight loss, and, mainly, cognitive impairment resulting in dementia, are highly prevalent [3].

The focus now shifts towards exploring MCI in this population, since it is considered that various interventions related to cognition in MCI could prove beneficial in terms of preventing dementia. Delving into the role of sleep on working memory capacity, this study aims to shed light into the intricate interplay between them in the population of older adults affected by MCI-related neurodegenerative mechanisms.

### 1.1. Mild Cognitive Impairment (MCI) in Older Adults 

Among all the possible syndromes that can occur in older adults, MCI can be distinguished as the most common. According to Anderson [4] and Tsolaki et al. [5], MCI affects from 10–15% to 24% of the population over the age of 65, being the stage between the anticipated decrease in memory and cognitive function associated with aging, and dementia [6]. The newly updated criteria for MCI necessitate (1) a noticeable change in cognition acknowledged by the individual or by observers; (2) a measurable decline in one or more cognitive domains; (3) the capability to perform daily activities independently; and (4) the lack of dementia [7]. 

Challenges in recalling events, as well as difficulties in orientation, planning, decision-making, and understanding instructions, are some of the symptoms that are prevalent in MCI patients. They may also exhibit one or multiple cognitive impairments in areas such as language, visuospatial skills, attention, and executive functioning, either with or without memory deficits [8]. Previous studies indicate that early deficits in visual episodic memory, executive function, semantic language/memory, attention, and working memory are highly indicative of the progression from MCI to AD [9].

### 1.2. The Role of Sleep and Sleep Deprivation in Cognition and, Especially, in Working Memory Capacity

In recent years, researchers have shown great interest in understanding how sleep and lack of sleep impact the physiological functioning of the human body, particularly the brain structures responsible for cognitive processes [10,11]. Maintaining sufficient sleep is vital for people to uphold their cognitive functions while they are awake, especially when involved in everyday activities such as studying and working [12,13].

Conversely, an extensive body of prior research has demonstrated the adverse effects that can occur from sleep deprivation on mental health [14,15,16]. Sleep deprivation is defined as the condition of getting less sleep than an individual’s usual need [17]. The most common subtypes are “total sleep deprivation” and “partial sleep deprivation”, in which the person loses a whole night of sleep or remains awake for several hours (usually more than 4 h) during the night, respectively [18]. In general, sleep deprivation has been linked to impairments in working memory function, attention, reaction time, accuracy, and omission rates [19]. The results of Martínez-Cancino et al. [20] suggest that the frontal lobes, recognized for their involvement in working memory functions, are profoundly affected following total sleep deprivation. This is evident in the form of decreased accuracy and increased reaction times, indicating a significant impairment in cognitive performance.

Although the general understanding of working memory capacity, as proposed by Baddeley and Hitch in 1974 [21], is that it functions as a system or mechanism for retaining task-relevant information while performing cognitive tasks, it remains challenging to provide a straightforward definition of exactly what working memory entails. In general, working memory refers to the ability of holding and manipulating information in the mind [22]. Working memory has a restricted storage capacity, meaning it can only hold a limited amount of information at a time. Additionally, the retention of information in working memory is temporary and lasts for a short duration before it is forgotten or replaced [23]. The role of working memory involves storing limited information in a readily accessible state. It supports planning, comprehension, reasoning, and problem-solving activities [24]. The widely accepted model, that is Baddeley’s model for working memory capacity, includes four components: the central executive (which coordinates the other three components), the phonological loop (responsible for storing auditory information), the visuospatial sketchpad (for maintaining visual and spatial information), and the episodic buffer, which combines separate pieces of information into larger units and binds information from different sources [25]. The episodic buffer is a passive mechanism for integrating information from various dimensions and sources and making it accessible to conscious awareness [26]. Additionally, it plays a crucial role in sustaining the bound representations of stimuli within working memory [27]. Recent research on working memory supports the idea that the WM updating component and the binding processes of the newest addition to Baddeley’s theory, that is, the conceptual construct of the episodic buffer, are interrelated [28].

There is no strong evidence as regards WM storage decrements in MCI [29,30,31,32,33]. On the other hand, a recent review article by Chehrehnegar et al. [34] reports that studies that used the digit span backwards task found lower processing scores in MCI patients in contrast to the control group. During a visual-spatial working memory task, they also had difficulty in processing and manipulating spatial information [35].

The decline in working memory abilities accompanied by significant changes in brain activity implies that individuals with MCI display weaknesses in various cognitive functions, such as distinguishing and categorizing stimuli, as well as updating and manipulation of information stored in working memory [36]. More recent scientific data indicate that working memory updating declines with age [37], which is more prevalent in MCI adults rather than in healthy older adults [38]. Typically, MCI patients encounter dysfunction in working memory updating and visuospatial attention, proved from studies using the N-back task [39,40]. This specific decline is observed due to the reduced activation of frontal lobes [39,41]. The latest research indicates that MCI patients who encounter problems in the working memory binding processes are more likely to develop AD [42], while they are prone to higher binding errors compared to healthy adults [43].

### 1.3. Sleep Changes, Time of Day, and Cognitive Functioning in Aging

It is already known that human aging is accompanied by different sleep patterns, compared to what happened before. Specifically, older adults sleep less than their younger counterparts, which has negative consequences for their cognitive ability [44,45]. Therefore, maintaining the duration of sleep is more important for older adults than for younger ones to mitigate the adverse effects on cognition caused by less sleep, and thus the compounding outcomes of aging. Moreover, since older adults typically get fewer hours of sleep, the beneficial role of night sleep on cognition may be limited in terms of time of day (ToD) conditions as well, compared to those in younger adults [44]. Seeing it from the inverse side, the primary focus of the majority of sleep research is to examine the effects of sleep deprivation on the human body and its functions, rather than delving into the benefits of sleep. Research studies have begun to explore this aspect by incorporating the ToD variable. ToD condition is commonly used in studies examining performance efficiency, with participants carrying out the same task at different times throughout the day [46]. The main advantage of this protocol is the fact that the participants’ sleep–wake cycle is uninterrupted. Additionally, studies using the ToD condition have proven useful for assessing cognitive performance because the procedure takes place in a natural and everyday environment [46]. The effects of ToD on cognitive performance have been examined in studies including sleep deprivation as an added parameter. Previous studies have investigated the relationship between task performance and sleep deprivation with assessments taking place at different hours during the day [47] or by explaining their interaction [48]. In particular, both ToD and sleep deprivation factors negatively impact cognitive performance, but only their combination is detrimental. Lower cognitive performance is observed when an extended period of wakefulness and circadian disorganization in terms of ToD occur together [48].

In general, our cognitive performance fluctuates throughout the day based on how well it aligns with our circadian rhythms, which is referred to as ToD preference, and therefore, individuals do not perform with equal effectiveness at all times of the day. In other words, there are certain times of the day that are better than others for performing cognitively demanding tasks [49]. Specifically, due to individual differences, some people prefer morning ToD for tasks related to attention, WM, inhibition, and decision-making, and are characterized as “morning types”, while others are “evening types” because the preferred ToD for the aforementioned cognitive aspects is in the afternoon [49]. The people pertaining to the first type (which are usually older adults) tend to have an earlier wake-up and bedtime, and typically exhibit higher levels of activity in the morning compared to others—the evening types—who prefer to wake up and go to sleep later, and experience peak performance during the later hours of the evening [50,51,52]. Previous scientific data indicated that there is a reduced performance during the day in highly demanding WM tasks for the “morning types”, while better performance observed in the “evening types” [44]. A change in the preferred ToD, which occurs as we get older, leads older adults to consider themselves as “morning types” who have better performance in the morning hours in more difficult cognitive processing tasks [53,54,55]. For example, the literature body indicates worse performance for the “morning types” in recall of prose texts, which are considered as particularly difficult tasks for WM [56]. Generally speaking, it is well known from past studies that older adults appear more vulnerable by the afternoon ToD condition in tasks including cognitive processing, divided attention, inhibitory control, emotion recognition, working memory, and verbal fluency [44,57,58,59].

Except for self-reports and task performance, the fact that older adults are categorized as “morning types” is accompanied by age-related physiological changes. Specifically, the circadian rhythms of older adults result in an earlier phase shift toward a morning chronotype and reduced amplitude [60,61]. Additionally, in aging there is an alteration in the production of hormones such as melatonin and reduced circadian melatonin rhythm aptitude, which indicates a slowing biological clock as the organism ages [62,63]. Moreover, studies on molecular rhythms in the aging brain have discovered a group of genome-wide transcripts that begin to exhibit circadian rhythm in expression in the prefrontal cortex exclusively in older adults, potentially representing a compensatory mechanism for the deregulation of the biological clock [64]. However, 1000 genes exhibit alterations in rhythmicity with advancing age, potentially contributing to altered sleep patterns and cognition [65].

Consequently, based on the aforementioned, there is evidence supporting the relationship between altered sleep, changes in preferred ToD, and cognitive performance in aging, neuropsychologically and physiologically.

### 1.4. Sleep in MCI Patients

According to Da Silva [66] the close monitoring of individuals with sleep disruptions is necessary to detect the early indications of dementia. Therefore, it is important to recognize and address sleep problems to maintain cognitive functioning, especially in individuals with MCI who experience both disturbances in their sleep and deficits in their cognition.

Research results indicate that patients with MCI have significant differences in their sleep patterns compared to healthy individuals. Indicative differences include more wakefulness after falling asleep, reduced overall sleep duration, delayed rapid eye movement sleep (REM), and reduced REM sleep [67]. In the same vein, Basta et al. [68] found that older MCI patients with insomnia or short sleep duration have increased cortisol levels compared to the cognitively non-insomnia participants of the study.

Considering both the potential detrimental effects of sleep disturbances on cognition in MCI, and the generalized decrements in specific working memory components in MCI, we examined whether the already low level of main WM components in MCI, as compared to healthy aging, could be lowered even more due to partial sleep deprivation conditions that the MCI patients may experience in their everyday life. Such an experience could be generated by the combination of sleep and ToD conditions (e.g., when an MCI patient must perform cognitive work in the afternoon, without taking a nap).

### 1.5. The Aim of This Study

This study aimed to examine whether the combination of sleep in MCI and afternoon ToD condition (that is, a condition with a long distance from nighttime sleep, without any nap in between) impacts working memory capacity as storage, processing, and updating in older adults with MCI. The hypothesis of the study was formulated as follows. Hypothesis 1: MCI patients would exhibit higher working memory storage, processing, and updating performance in the morning and immediately after night sleep, than in the afternoon, after many hours of being awake. 

## 2. Methods

### 2.1. Design

The study followed a within-subjects design, wherein the same participants underwent two separate evaluations: first condition: “early in the morning and immediately after waking up from night sleep”, vs. second condition: “in the afternoon, after many hours since night sleep without any nap in between”. The two assessments were spaced 1 week apart to mitigate any potential mnemonic biases. Before and during each phase of the study, participants refrained from consuming caffeine and alcohol for a span of 72 h. They maintained a regular sleep–wake schedule, with the average sleep duration ranging from 5 to 7 h. It was explicitly prohibited for participants to take any intervening naps before the afternoon examination. The participants were assigned to each experimental condition in such a way that half first completed the task in the morning, while the other half started in the afternoon. Given the within-subjects design, we aimed for a minimum sample size of *n* ≥ 45 based on G*Power for Windows (version 3.1.9.7) [69].

### 2.2. Participants

Fifty (50) ΜCI patients, aged 65 years or older [Ν = 50, 21 men (42%) and 29 women (58%), age range: 65 to 81 years, M = 70.28 years, SD = 4.23 years, educational level range: 6 to 20 years of schooling, M = 11.54, SD = 3.78, Montreal Cognitive Assessment (MoCA) score range: 23 to 29, M = 25.02, SD = 1.76] were recruited by community workers via an MCI patients list provided by the Alzheimer’s Hellas Day Care Unit “Saint Ioannis” in Thessaloniki and the Greek Alzheimer’s Association of Larissa. The patients were participants of the cognitive training programs during the period of May 2023 to April 2024.

For their diagnosis, the criteria of the Diagnostic and Statistical Manual of Mental Disorders, Fifth Edition (DSM 5) for mild neurocognitive disorders were used, supported by a range of assessments, including neurological examination, neuropsychological and neuropsychiatric evaluations, neuroimaging (either computed tomography or magnetic resonance imaging), and blood tests [70]. Inclusion criteria encompassed (1) meeting the DSM-5 criteria for Minor Neurocognitive Disorders, (2) achieving a MoCA total score ≥ 23, (3) reaching stage 3 on the Global Deterioration Scale, and (4) scoring at least 1.5 standard deviations (SD) below the normal mean for age and educational level in at least one cognitive domain as per the administered neuropsychological tests. All the participants had an MCI diagnosis for at least 2 years.

### 2.3. Ethical Standards

The research methodology received approval from the Scientific and Ethics Committee of the Alzheimer’s Hellas, adhering to the guidelines set forth in the Helsinki Declaration with code reference 84/14-12-2022. Following the General Data Protection Regulation (GDPR), every participant was thoroughly briefed on the procedure and purpose of the study, and their informed consent was obtained in written form. They were given the chance to pose questions and were assured that their data would be collected confidentially in an electronic database. Their involvement was voluntary, and they were informed that they could withdraw at any point without any consequences.

### 2.4. Procedure

The two experimental conditions took place in a place of the participant’s choice. Some of them opted to complete the assessment at the Alzheimer’s Hellas or Alzheimer’s Association of Larissa, while others decided to do so at their home. In each instance, a quiet room was selected, to minimize distractions. The precise ToD for the two assessments was determined through direct communication with each participant. Specifically, for the first condition (“early in the morning and after night sleep”), the participants engaged in a working memory test around 8–9 a.m. (±30 min), approximately 2 h after waking up. In the case of the second condition (“in the afternoon and after many hours since night sleep”), the same test was administered to the participants at around 3:00 p.m. (±30 min), on average, 9.5 h after awakening. For both conditions, each participant completed the task individually. Keeping in mind that all patients were participants in cognitive training programs of the day care units, it is worth mentioning that on the day of the afternoon assessment, they remained at these facilities the entire time, avoiding any consumption of caffeine and alcohol, and any attempts to nap were monitored. On the other hand, it is difficult to determine the exact duration that the patients slept, because the researcher was not in their homes. The main advantage of this experimental design is that the environment was familiar to them, and therefore, this study has strong ecological validity.

### 2.5. Instrument

The R4Alz battery was developed [37,38,71] to differentiate cognitive control ability of older cognitively healthy people from people with Subjective Cognitive Decline (SCD) and older adults diagnosed with MCI. In this study, the computerized form (http://r4alz-online.issel.ee.auth.gr/) (accessed on 15 December 2022) was used to examine only working memory capacity. Using the first task [WM Capacity & Updating Task (WMCUT)], which is part of this broader battery, we assessed three working memory components.

Specifically, the WMCUT comprises 3 subtasks: (a) a working memory storage subtask (WMCUT-Subtask 1), (b) a working memory processing subtask (WMCUT-Subtask 2), and (c) a working memory updating subtask, enriched by episodic buffer recruitment (WMCUT-Subtask 3). Each subtask includes 6 conditions, i.e., 18 in total. Subtask 1 (working memory storage) includes a sequence of between two (2) and seven (7) virtual pads in which sequentially a white light appears when activated. The participant is instructed to memorize the order in which these lights are turned on and then to indicate the correct sequence, moving the computer mouse over and clicking on each virtual pad. The participant’s performance is measured by the number of lights correctly remembered and indicated in the correct sequence, ranging from a minimum score of two (2) to a maximum score of seven (7) [37].

The next subtask (subtask 2—working memory processing) follows the same pattern as for the procedure and score, but this time the participant is required to remember and indicate the sequence of the lights from the last to the first one [37].

Subtask 3 (working memory updating and episodic buffer) consists of six conditions (3a–3f) of increasing difficulty, with minimum score = zero (0) and maximum score = fourteen (14). The score range for 3a and 3b conditions is zero (0) (minimum score) to one (1) (maximum score). Concerning the conditions 3c and 3d, the minimum score is also zero (0), but the maximum score is two (2). The 3e condition is scored from zero (0) to three (3), and the 3f score ranges from zero (0) to five (5). All scores are in integer form [37]. The six conditions are presented in detail below:

- The first condition (3a) includes seven (7) virtual pads, i.e., six (6) white lights and one (1) green, which are lighted one after the other, while the participant has been given the instruction to remember only the location of the green light. Then, the lights turn off and light on again one by one. The participant is required to detect and click, with the computer’s mouse, the green light if it appears to the right of its initial position. The correct answer scores one (1) point, while the wrong answer scores zero (0) points.
- The same procedure is followed in this condition as well, but here the six (6) white lights are replaced with different colors (white, red, blue, magenta, cyan, and yellow). The participant must remember and click with the computer’s mouse the green light only if it appears two (2) places to the right of its initial position. The score procedure in this condition remains the same.
- The 3c condition follows the same line as the previous one, but now the participant needs to remember both the place of green and red lights and click on the virtual pads in that case that the lights appear two (2) places to the right of their initial position. The maximum score here is two (2), if the participant correctly detects the place of both lights, one (1) if they detect only one, and zero (0) for no correct detections.
- The next condition follows the same pattern, but the participant is required to detect and click on the green light only if it appears two (2) places to the right of its initial position, and the red light only if it appears two (2) places to the left of its initial position. Scoring here remains the same.
- This condition consists of only green and red lights, while the participant is instructed to remember the last position where a green light appeared. This position changes continuously throughout the task and the participant needs to click on each green light if it appears two (2) positions to the right of where the last green light appeared. This condition includes seven (7) red and eight (8) green lights, of which three (3) are correct.
- The last condition also follows the same pattern. The only difference here is that the participant must remember the last position and click on both green and red lights every time they appear two (2) places to the right from where the last green and red lights appear. There are seven (7) red lights and eight (8) green ones, from which five (5) are correct [37].

**Figure 1:**
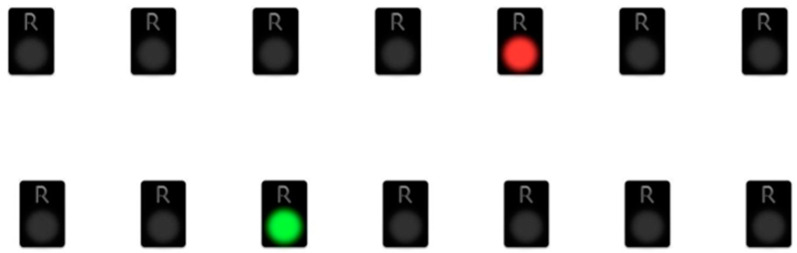
An indicative task condition.

All lights’ colors were selected to be easily discernible for participants, minimizing any potential confusion. The administration of the WMCUT usually requires 15 to 20 min, varying based on the participant’s age and condition. The WMCUT was conducted following the established protocol provided in the appropriate manual. To acquaint the participant with the task’s demands, a practice item was presented before the primary task as shown in Figure 1 [37].

### 2.6. Statistical Analysis

For data analysis, IBM SPSS Statistics for Windows (version 23.0; IBM Corp, Armonk, NY, USA) was used. Repeated measures analyses of variance were applied to the data. The within-subjects variable of the ToD condition had two levels: “early in the morning and after night sleep” vs. “in the afternoon and after many hours since night sleep”. Partial eta-squared (η^2^) was used for the estimation of the effect size, while statistical significance (*p*) was defined at the 0.05 level.

## 3. Results

**Figure 2:**
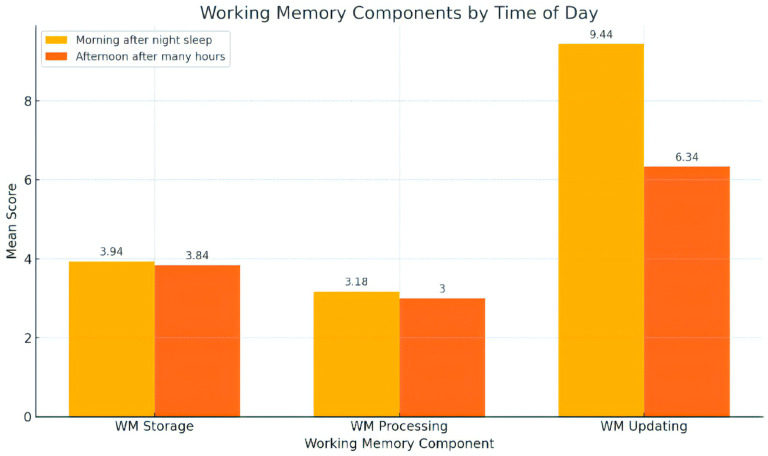
Working memory components by time of day.

MCI patients’ performance as regards the three subtests examining the three different components of WM capacity is shown in Table 1 and Figure 2.

The application of repeated measures analysis of variance to the data indicates that there was not a significant effect of condition (ToD) type on working memory storage (F (1, 49) = 0.754, *p* = 0.389, η^2^ = 0.015). Likewise, no statistically significant difference was found between the performance in the two conditions (ToDs) for the working memory processing component (F (1, 49) = 1.981, *p* = 0.172, η^2^ = 0.038). On the other hand, a robust statistically significant effect of condition (ToD) type on working memory updating was found (F (1, 49) = 117.429, *p* < 0.001, η^2^ = 0.706). The results showed that WM updating performance decreased in the afternoon condition compared to the morning condition (see Table 1).

In our analysis, we tested the assumption of sphericity using Mauchly’s test. The test indicated that the assumption of sphericity was satisfied for all variables (Mauchly’s W = 1, *p* =< 0.001 for WM storage, W= 1, *p* =< 0.001 for WM processing, and W = 1, *p* =< 0.001 for WM updating). Therefore, no correction was necessary for the degrees of freedom in our analyses.

## 4. Discussion

The study aimed at examining the potential mixed effects of sleep and afternoon ToD condition on working memory storage, processing, and updating in MCI patients aged over 65 years old. The prevalent cognitive challenges in MCI often involve the loss of episodic memory and difficulties in working memory capacity, which play a vital role in learning, information processing, executive functioning, and comprehension [72,73,74]. For this purpose, a within-subjects experiment was designed in which the same MCI participants were examined twice, i.e., “in the morning and after night sleep” and “in the afternoon and after many hours of being awake”, to assess their working memory capacity. The primary advantages of the study were (a) the use of a novel diagnostic tool for neurocognitive disorders to discriminate the differences in working memory functioning of MCI patients between the two conditions, and (b) the within-subjects design of the study, which ensured that main confounding variables were not involved. The results clearly indicate that older adults with MCI have a significantly higher-level performance in working memory updating in the morning and immediately after night sleep, than in the afternoon and after many hours of being awake. Therefore, it is clearly demonstrated that their WM updating ability is negatively affected by the combined effects of sleep disturbances in MCI and the afternoon ToD condition. Because the participants did not show any statistically significant difference regarding WM storage and WM processing between the two conditions, Hypothesis 1 is partially confirmed.

Some studies have already described that the extended period of several hours following a night’s sleep, without an intervening nap, can serve as a condition of sleep deprivation for older adults with multiple cognitive disadvantages [19,75]. According to the present findings, MCI patients appear more susceptible regarding the decrease in their performance in the afternoon in tasks that are more cognitively demanding to execute. The main reasons supporting the complexity of the WM updating subtask (WMCUT-S3) are the combination of cognitive domains involved and the additional cognitive component of the “episodic buffer”. While the subtasks for assessing WM storage and WM processing mainly evaluate visuospatial working memory, the WM updating subtask also requires high WM memory load level, perception, and binding processes [38,76], especially in the last two conditions due to the unforeseen lights’ activation and different locations of the virtual pads.

Previous research mentioned that the updating process of working memory engages cognitive functions such as attention [76,77] and encoding of new information [78,79]. Indeed, Artuso et al. [76] found that cognitive decline through age is due to the impaired function of updating functions, stating that binding-updating tasks’ conditions with both high and low memory load are too challenging. Given that the WM updating subcomponent task includes conditions for assessing the episodic buffer [37], it engages binding-related functions [80], which are assisted by executive control, attention, and fluid intelligence in general [28]. Actually, the binding function is related to visual WM [28] and requires attention for preventing disruption from competing stimuli [77], which is low after sleep deprivation [81]. Equally, Matysiak et al. [82] observed that working memory updating demands a high level of visual attentional resources, which, however, are reduced after sleep loss [83]. To elaborate, previous scientific data have shown that there is an alteration in the left visual field of the right hemisphere after sleep deprivation, reflecting abnormalities in visuospatial perception [84]. Finally, a study from Sullan et al. [85] shows poor detection ability and visual processing after total sleep deprivation, which is explained by decreased activation of frontoparietal areas responsible for managing top-down attentional control [86].

In respect to the neural networks, which are responsible for the episodic buffer, the literature highlights the role of the left anterior hippocampus [87]. In addition, recent studies have found that the medial temporal lobe and the temporal-parietal-frontal areas also contribute to the episodic buffer [80]. These brain areas have been proven to be vulnerable to sleep loss, which is evident from the changes in molecular signaling in the hippocampus—which plays a significant role in synaptic plasticity [88]—and the aggravation of neuropathological mechanisms related to the medial temporal lobe [89].

The binding updating process is involved in tasks where participants need to combine objects’ features like shape color or location [28], a parameter that the WM updating task incorporates through the various locations and colors of the virtual pads. Specifically, in the WM updating subtask, the participants are required to remember the last position of each virtual pad (of red or green color), a cognitive ability in which maintenance of bound object representations in WM with undisrupted attention is needed [28]. As already mentioned, sleep deprivation has a negative impact on high-level attentional resources, indicating that the binding process of the episodic buffer is affected as well. This is evident by decreased activation of the hippocampus in sleep-deprived individuals, which is responsible for binding, retrieval, and encoding of items [90]. In the same way, a study which investigated the neural networks involved when people are required to maintain information during a working memory updating task, ascertained that decreased P3 amplitude after total sleep deprivation was related to decreased working memory updating [83].

Although the combination of sleep and ToD conditions has an impact on working memory updating in MCI patients, the results of the study clearly show that working memory processing and storage are almost unaffected by the condition “afternoon and after many hours of being awake”. This finding, at least as regards working memory processing, seems to not be in line with previous research, which revealed that lack of sleep can lead to impairment [91,92]. Similar data can be observed in the study of Blair et al. [93], who indicated that the decline in the accuracy of working memory processing is anticipated with age due to reduced inhibition. The most possible explanation for this discrepancy may be that the afternoon condition affects older MCI patients in tasks characterized by higher difficulty in terms of multilevel cognitive demands, while in simpler tasks in terms of the number and combinatory function of cognitive abilities recruited, such as the working memory processing and storage tasks of the R4Alz tool, their performance remains the same irrespective of sleep deprivation-like conditions experienced in everyday life. Considering that SCD is a previous stage of MCI, these findings could potentially apply not only to MCI patients, but also to people with SCD under sleep deprivation conditions and to dementia patients as well.

## 5. Conclusions

Considering all the above, it can be suggested that the mixed sleep and afternoon ToD effects on cognition seem to be revealed in MCI, when the tasks are more cognitively demanding. At a deeper level, this could imply that the brain areas associated with demanding tasks such as working memory updating, including the frontoparietal network (FPN), basal ganglia, thalamus [94], and hippocampus [87], exhibit reduced activity during these conditions in MCI patients. Given that good sleep quality can improve the connectivity and the activity of the FPN in these patients [95,96], it is evident that such conditions can disrupt this brain network. Keeping in mind that FPN is involved not only in the working memory, but also in goal-oriented attention, inhibition, and adaptation to feedback, we can assume that sleep in MCI and afternoon ToD negatively affect these additional functions or their combination in demanding tasks [97].

## 6. Limitations and Further Research

While participants were instructed to refrain from consuming coffee and alcohol for a period of 72 h, it is important to note that adherence to this instruction was not verified through laboratory testing, thus introducing a potential uncertainty regarding the sleep deprivation-like condition (“in the afternoon and after many hours of being awake”). Replication of these findings in a sample of more day care units and hospitals, by employing objective measures of sleep and neuroimaging techniques, would further strengthen the validity and reliability of these findings. Furthermore, it is recommended to develop more intricate experimental designs that encompass varying sleep conditions. This approach would identify strategies for assisting older adults in sustaining a heightened level of working memory ability throughout the entirety of the day.

## 7. Applications in Clinical Practice and Everyday Life

This research highlights the importance of sleep in MCI patients when engaged in daily activities that demand sustained attention, executive functions, and binding processes to update information (e.g., working memory), such as driving and working. In cases where MCI older adults need to arrange responsibilities or execute important tasks, it may be more preferable to do so in the morning and after a night’s sleep, rather than in the afternoon after many hours of being awake and tired. Hence, good sleep and stable sleep patterns are necessary for maintaining high working memory capacity. At the same time, tasks enhancing working memory components have been proven to be useful in MCI patients, and, therefore, cognitive rehabilitation programs could be more beneficial for them in the morning hours than in the afternoon. Daytime napping could also be helpful for patients with MCI to improve their cognitive function. In fact, 20-min naps taken between 1:00 p.m. and 3:00 p.m. have been shown to have positive effects on cognition in these patients [98], while previous data demonstrated that working memory can be improved by daytime sleep [99]. Therefore, napping during the day can be considered an important intervention method for moderating the adverse effects of sleep deprivation [100].

## Figures and Tables

**Table 1:** MCI patients’ mean and standard deviation for working memory storage, processing, and updating in the two conditions of the study.

Working Memory Components’ Mean Scores	Morning after Night Sleep					Afternoon after Many Hours Since Night Sleep		
	M	SD	Minimum	Maximum	M	SD	Minimum	Maximum
WM storage	3.94	0.73	2	6	3.84	0.71	2	6
WM processing	3.18	0.71	1	4	3.00	0.67	1	4
WM updating *	9.44	2.7	2	14	6.34	2.9	1	14

* *p* < 0.001.

## Data Availability

The data presented in this study are available on request from the corresponding author due to privacy reasons.
